# An updated evidence-informed framework for dental sedation: drugs, routes, patient selection, monitoring, and emergency preparedness

**DOI:** 10.3389/froh.2026.1915718

**Published:** 2026-09-16

**Authors:** Giath Gazal, Mohammad Zakaria Nassani, Sam Thomas Kuriadom, Kusai Baroudi

**Affiliations:** 1 Hillside Dental Practice, Manx Care, Nobles Hospital, Isle of Man, United Kingdom; 2 Department of Oral and Maxillofacial Surgery, Faculty of Dentistry, Aleppo University, Aleppo, Syria; 3 Department of Restorative and Prosthetic Dental Sciences, College of Dentistry, Dar Al Uloom University, Riyadh, Saudi Arabia; 4 Department of Clinical Sciences, College of Dentistry, Ajman University, Ajman, United Arab Emirates; 5 Pos Graduation Program in Health Sciences, Department of Dentistry, University of Taubaté (UNITAU), Taubaté, Brazil

**Keywords:** conscious sedation, dental anxiety, dental sedation, emergency preparedness, midazolam, monitoring, nitrous oxide, patient safety

## Abstract

**Background:**

Dental sedation facilitates care for patients with anxiety, behavioral challenges, pronounced gag reflexes, special healthcare needs, or complex treatment requirements. This review aimed to integrate current evidence into a clinically applicable framework for individualized sedation planning in dental practice.

**Methods:**

An evidence-informed narrative review was conducted using PubMed/MEDLINE, Scopus, Web of Science, and the Cochrane Library, covering primarily January 2014 to September 2025. Literature identification and selection were reported with reference to PRISMA 2020 principles. Clinical guidelines, systematic reviews and meta-analyses, randomized controlled trials, observational studies, and relevant clinical literature were synthesized narratively and organized descriptively according to study design and their principal role in the narrative synthesis.

**Results:**

Evidence supports individualized sedation selection based on patient age, medical and ASA status, airway risk, anxiety and cooperation, procedural requirements, intended sedation depth, clinician competence, and available monitoring and rescue resources. Nitrous oxide-oxygen and midazolam-based techniques are supported for appropriately selected patients requiring minimal-to-moderate sedation. Advanced or deep sedation requires appropriately trained and credentialed personnel, appropriate physiological monitoring, and immediate airway-rescue capability. Dexmedetomidine- and ketamine-based regimens may provide specialized options in selected populations, although protocols and applicability remain heterogeneous. The evidence was integrated into a conceptual framework addressing patient assessment, pharmacological options, monitoring, recovery, and emergency preparedness.

**Conclusions:**

No single sedative drug or administration route is universally optimal for all dental patients. The available evidence supports individualized sedation selection based on patient characteristics, procedural requirements, intended sedation depth, clinician competence, and available monitoring and rescue resources. Nitrous oxide–oxygen and midazolam-based techniques remain well-supported options for appropriately selected patients, whereas advanced or deep sedation should be reserved for appropriately equipped settings with trained and credentialed personnel.

## Introduction

Dental sedation is a cornerstone of modern clinical practice, designed to facilitate treatment in patients who would otherwise be unable to tolerate dental procedures due to fear, anxiety, medical comorbidities, or behavioural challenges (1–3). It involves the careful use of pharmacological agents to depress the central nervous system (CNS) in a controlled and reversible manner, while maintaining verbal contact, protective reflexes, and stable vital signs. Sedation in dentistry exists on a continuum, ranging from minimal sedation (anxiolysis) through moderate sedation (conscious sedation), to deep sedation approaching general anaesthesia (4). Moderate sedation (conscious sedation) is widely used in dental practice because it can provide anxiolysis and amnesia while maintaining purposeful responsiveness; however, airway and ventilatory function must be continuously assessed according to the intended and reasonably foreseeable depth of sedation. Sedation is indicated in a wide spectrum of patients. In adults, it is valuable for those with severe dental phobia, pronounced gag reflex, mild-to-moderate learning disabilities, or movement disorders (5). In paediatric dentistry, sedation is critical for very young children unable to cooperate, for anxious children, and for those requiring extensive procedures such as pulp therapy and restorations (6, 7). Patients with special care needs, including individuals with cognitive impairment or physical disability, may also benefit from tailored sedation strategies. Suitability depends on medical status: American Society of Anesthesiologists (ASA) Physical Status Classification I–II patients and those with well-controlled systemic disease (e.g., stable asthma, diabetes mellitus, or hypertension) can safely undergo sedation in outpatient or community settings (8). Patients with significant or unstable systemic disease, including selected ASA III–IV patients, require individualized risk assessment and may require consultation, modification of the sedation plan, or management in an appropriately equipped hospital or specialist setting, depending on medical stability, intended sedation depth, procedural complexity, provider competence, and available monitoring and rescue resources. Children with active respiratory tract infections require careful assessment, and elective sedation may need to be postponed when respiratory or airway risk is increased (9, 10).

Benzodiazepines, particularly midazolam and the newer remimazolam, remain the most widely used dental sedatives. Oral midazolam is commonly employed for young children requiring rapid, short-duration care, while intravenous (IV) administration is standard for adults (11). Nitrous oxide–oxygen inhalation is a widely used minimal-sedation technique with a favorable safety profile in appropriately selected pediatric patients (12). Intranasal midazolam is generally recommended for children aged 4–6 years, with evidence suggesting that higher doses (0.5 mg/kg) are more effective at reducing anxiety and improving behaviour compared to lower doses (0.3 mg/kg) (13). Oral midazolam, while also safe and effective, is typically used for children between 5 and 10 years of age who require a longer working time (14). Deep sedation may involve combinations such as midazolam with ketamine, dexmedetomidine, or propofol, but such regimens require a trained sedationist or anaesthetist because of the potential for respiratory depression and haemodynamic instability (15–17). Ketamine is particularly useful due to its unique dissociative properties, providing analgesia and amnesia without significant risk of hypotension (18). Dexmedetomidine, an alpha-2 adrenergic agonist, induces sedation resembling natural sleep while offering prolonged analgesia, anxiolysis, and temperature reduction (19). Propofol, a GABA-A receptor-modulating intravenous sedative-hypnotic, is valued for its rapid onset, titratability, and rapid recovery, but requires appropriate expertise and airway-rescue capability because of its potential for respiratory and cardiovascular depression (20). The safe delivery of these agents demands thorough knowledge of pharmacology, awareness of drug–drug interactions, and immediate access to emergency resuscitative drugs and equipment (21). At a mechanistic level, dental sedatives act on specific receptor systems to reduce anxiety, dampen exaggerated protective reflexes, and improve patient tolerance of treatment. Benzodiazepines enhance inhibitory neurotransmission via gamma-aminobutyric acid type A (GABA-A) receptors, producing anxiolysis, sedation, and anterograde amnesia (22). Nitrous oxide exerts effects through N-methyl-D-aspartate (NMDA) receptor antagonism and endogenous opioid release, providing relaxation and mild analgesia (23). Agents such as ketamine, dexmedetomidine, and propofol act through NMDA antagonism, alpha-2 adrenergic agonism, and GABAergic potentiation, respectively, enabling their use in deep sedation when appropriately monitored (24, 25). By modulating these neurochemical pathways, sedatives suppress fear and gag reflexes while preserving airway protective reflexes.

This comprehensive review aims to answer the research question: “How can current evidence on sedative agents, administration routes, patient characteristics, monitoring, and emergency preparedness inform individualized sedation planning in dental practice?” The review provides an evidence-based framework for dental practitioners, integrating pharmacological profiles, administration protocols, and patient selection criteria. Furthermore, it introduces an evidence-informed conceptual framework for sedation selection, ranging from minimal to advanced sedation approaches according to individualized patient assessment, procedural requirements, provider competence, monitoring and rescue capabilities, and applicable professional and regulatory requirements.

## Methods

### Review design and search strategy

This structured, evidence-informed narrative review was designed to synthesize clinically relevant evidence concerning patient assessment, pharmacological sedation, monitoring, recovery, and emergency preparedness in dental practice. It was conducted as a narrative evidence synthesis rather than a systematic review or meta-analysis. A structured literature search and study-selection process was used to enhance transparency and reproducibility. PRISMA 2020 principles were used solely to report literature identification, screening, eligibility, and inclusion and should not be interpreted as indicating that the review fulfilled all methodological requirements of a systematic review (26). The literature-selection process is summarized in Figure 1. No review protocol was prospectively registered. A structured search of PubMed/MEDLINE, Scopus, Web of Science, and the Cochrane Library was conducted for English-language literature published from January 2014 to September 2025, with earlier seminal sources retained selectively where necessary. Search concepts included dental sedation, conscious sedation, nitrous oxide, midazolam, dexmedetomidine, propofol, ketamine, remimazolam, anxiety, gag reflex, ASA status, airway assessment, monitoring, recovery, and emergency preparedness. Database-specific search syntax was adapted to the indexing requirements of each database. The complete search strategies, including Boolean operators, field tags, date limits, and the date of the final search, are provided in Supplementary Table S1.

**Figure 1:**
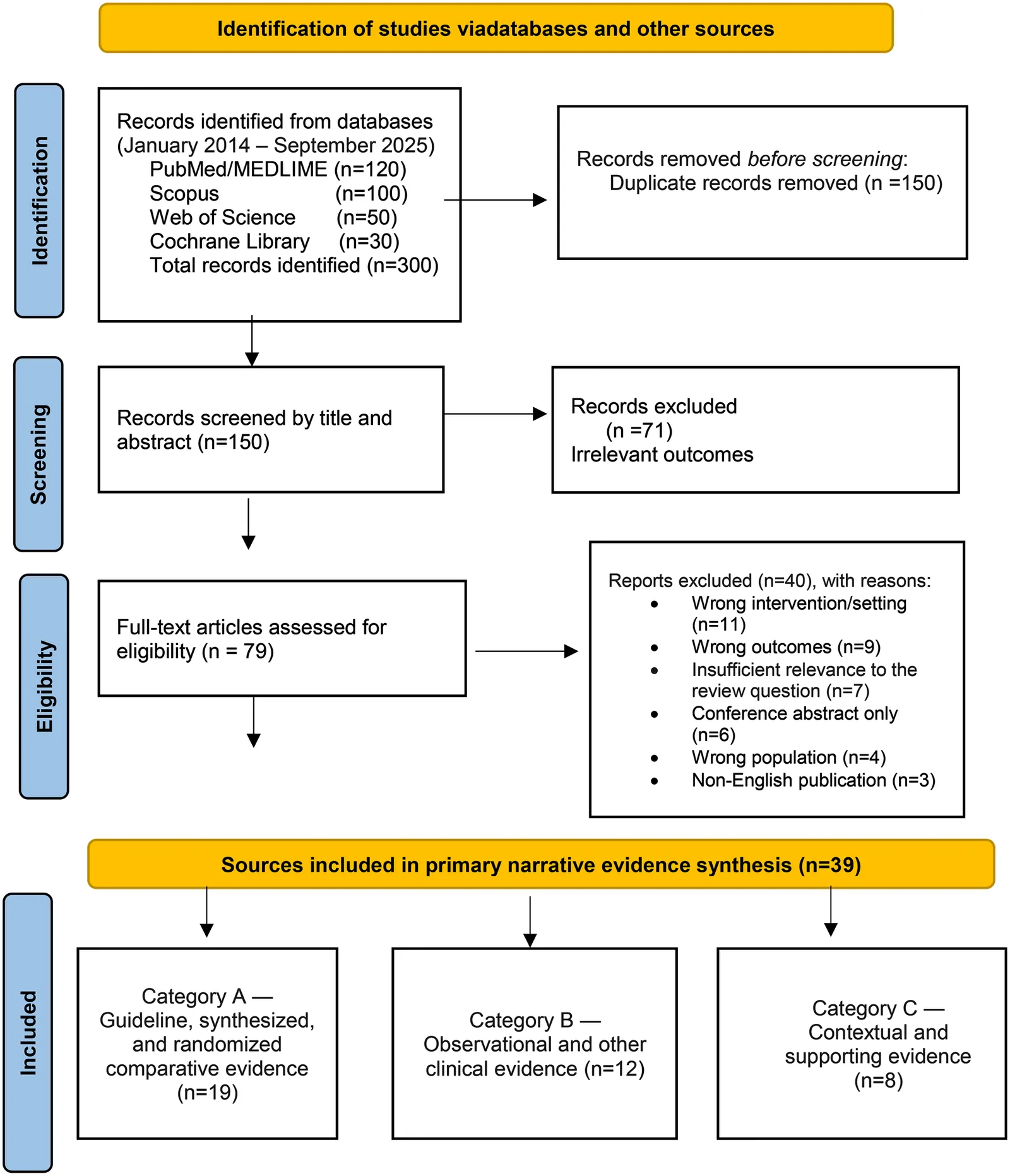
PRISMA 2020-based flow diagram illustrating literature identification, screening, eligibility assessment, and inclusion in the structured narrative review. PRISMA: preferred reporting items for systematic reviews and meta-analyses.

Eligibility and Study Selection: Eligible evidence included randomized controlled trials, systematic reviews and meta-analyses, observational and comparative clinical studies, clinical guidelines, and relevant clinical reviews addressing dental sedation or procedural-sedation principles directly applicable to dentistry. Sources were considered if they addressed patient assessment and selection, anxiety or behavioral management, sedative agents and routes, dosing considerations, sedation depth, physiological monitoring, airway safety, recovery, adverse events, reversal agents, or emergency preparedness. Non-dental procedural-sedation evidence was included selectively when relevant pharmacological, monitoring, airway-management, or rescue principles were applicable to dental sedation, considering differences in patient population, sedation depth, clinical setting, and provider expertise. Such evidence informed general safety and rescue principles rather than being considered directly equivalent to dental practice. Case reports, conference abstracts, animal or *in vitro* studies, non-English publications, and sources lacking sufficient clinical relevance were excluded from the primary evidence synthesis. Protocol-based, case-specific, or indirectly applicable publications considered useful for background interpretation were retained only as contextual sources and were not included in the primary evidence classification. Records were screened by title and abstract, followed by full-text assessment of potentially eligible publications. Duplicate and ineligible records were excluded, with reasons for full-text exclusion categorized where applicable. The identification, screening, eligibility, and inclusion process is presented in the PRISMA 2020 flow diagram (Figure 1) (26). All title/abstract screening and full-text eligibility decisions were performed manually by the authors; no automated screening or exclusion tools were used.

### Data synthesis and evidence classification

Relevant data were extracted on study design, patient population, sedation technique, pharmacological agent and route, dosing considerations, clinical outcomes, adverse events, monitoring, recovery, and emergency-management requirements. Because of substantial heterogeneity in populations, interventions, sedation protocols, and reported outcomes, quantitative meta-analysis was not undertaken; evidence was synthesized narratively. For descriptive organization of the narrative synthesis, sources were grouped pragmatically according to study design and their principal role in informing the review: Category A comprised authoritative clinical guidelines, systematic reviews/meta-analyses, and relevant randomized controlled trials; Category B comprised observational, retrospective, comparative non-randomized, and other clinical studies; and Category C comprised narrative or clinical reviews, foundational pharmacological evidence, and other indirect supporting literature. These categories describe the type and role of evidence and should not be interpreted as a linear ranking of methodological quality or certainty. Sources within the same category may differ substantially in methodological rigor, directness, population, and clinical applicability. The classification was not a formal GRADE certainty-of-evidence assessment and did not imply that all Category A sources represented uniformly high-certainty evidence. Sources used primarily for contextual, protocol-based, case-specific, or methodological purposes were considered separately from the primary evidence synthesis. The classification, distribution, and principal clinical contribution of the evidence supporting the proposed sedation framework are summarized in Table 1.

**Table 1:** Evidence-source classification and contribution to the narrative synthesis.

Evidence category	Evidence type	No. of sources	Principal role in the review
Category A—Guideline, synthesized, and randomized comparative evidence	Authoritative clinical guidelines; systematic reviews/meta-analyses; relevant randomized controlled trials	19	Clinical guidance; comparative efficacy and safety; monitoring, sedation, and major practice recommendations
Category B—Observational and other clinical evidence	Observational, retrospective, comparative non-randomized, and other clinically relevant studies	12	Clinical outcomes; real-world practice; adverse events; sedation regimens and implementation considerations
Category C — Contextual and supporting evidence	Narrative/clinical reviews; foundational pharmacological evidence; other indirect supporting literature	8	Pharmacological context, clinical interpretation, and supporting background
Total	—	39	Primary evidence synthesis

A–C categories are used descriptively to organize evidence sources according to study design and their role in the narrative synthesis; they do not represent a formal hierarchy of methodological quality or certainty. Sources within each category may vary in methodological rigor, directness, and clinical applicability. No formal GRADE certainty-of-evidence assessment was performed. Contextual, protocol-based, case-specific, and methodological sources considered outside the primary evidence synthesis are not included in these counts.

### Development of the sedation framework

The synthesized evidence was integrated into an evidence-informed conceptual framework for individualized sedation selection rather than a rigid or mandatory sequential escalation algorithm. Assessment tools such as the Modified Dental Anxiety Scale (MDAS), Frankl Behavior Rating Scale, and Gagging Severity Index (GSI) may support patient assessment but should not independently determine drug selection, route of administration, sedation depth, or escalation to deep sedation. Sedation decisions should incorporate patient age, medical and ASA status, airway characteristics and obstructive sleep apnea risk, medications and potential interactions, procedure complexity and duration, intended and foreseeable sedation depth, clinician competence, monitoring and rescue capabilities, facility resources, and applicable professional and regulatory requirements (16–20, 27). Advanced techniques, including propofol-based deep sedation, should be undertaken only in appropriately equipped settings by suitably trained and credentialed personnel, with monitoring appropriate to sedation depth, immediate airway-rescue and positive-pressure ventilation capability, emergency and resuscitation resources, and compliance with applicable professional and regulatory requirements (16–20, 27).

## Results

Evidence was organized according to the predefined descriptive evidence-source categories (Categories A–C), with the distribution and principal contributions of supporting sources summarized in Table 1. Given the heterogeneity in study design, patient populations, sedation techniques, dosing regimens, and reported outcomes, findings were synthesized narratively. Overall, the evidence supported an individualized approach to sedation selection based on comprehensive patient assessment, procedural requirements, intended sedation depth, provider competence, monitoring and rescue capabilities, and applicable professional guidance.

Table 2 summarizes clinical factors relevant to individualized sedation assessment and planning. Validated instruments such as the Modified Dental Anxiety Scale (MDAS), Frankl Behavior Rating Scale, and Gagging Severity Index (GSI) may assist in characterizing anxiety, cooperation, and gag-reflex severity; however, these measures should not be interpreted as stand-alone criteria for selecting a specific sedative agent or sedation depth. Sedation planning requires integration of patient age, medical and ASA status, airway risk, previous sedation experience, procedural complexity and duration, clinician competence, and available monitoring and rescue resources.

**Table 2:** Clinical factors supporting individualized sedation assessment and planning.

Clinical profile	Assessment considerations	Potential sedation considerations	Key decision factors
Mild anxiety; cooperative patient	MDAS, Frankl, GSI may support assessment	Behavioral techniques and/or nitrous oxide–oxygen may be considered	Age, cooperation, procedure, medical status
Moderate anxiety or limited cooperation	Anxiety/behavior scales used as supportive tools	Nitrous oxide, oral/intranasal sedation, or titratable IV sedation may be considered according to patient and setting	Airway, ASA status, age, procedure duration, provider competence
Severe anxiety, significant behavioral difficulty, or complex procedural needs	Comprehensive individualized assessment required	Advanced sedation may be considered when clinically justified	Medical/airway risk, intended depth, provider credentials, monitoring/rescue resources

MDAS, Frankl, and GSI scores are supportive assessment measures and should not be used in isolation to prescribe a specific sedative agent, route, or depth of sedation.

Table 3 summarizes the primary sedative agents, their routes, dosages, and key clinical considerations. Nitrous oxide–oxygen remains a well-supported option for minimal sedation in appropriately selected patients because of its rapid onset and recovery, titratability, and favorable safety profile (11, 28, 29). Midazolam, administered orally, intranasally, or intravenously, remains the cornerstone for pediatric and adult sedation, offering strong anxiolytic and amnestic properties (3, 4, 12, 14). Dexmedetomidine, alone or in combination with ketamine, is increasingly used in complex behavioral cases or patients with autism spectrum disorder, providing deep sedation with stable hemodynamics and minimal respiratory depression (30–33). Propofol is reserved for advanced sedation under anesthetist supervision, while remimazolam offers predictable recovery and reduced respiratory depression but is limited by cost and pediatric data (22, 23). Reversal agents such as flumazenil for benzodiazepine oversedation and naloxone for opioid-related complications enhance safety across all sedation scenarios (34–36).

**Table 3:** Sedative agents, routes, indicative dosing considerations, evidence categories, and key safety requirements.

Agent/combination	Route & dosage	Indications/clinical notes	Advantages	Disadvantages/precautions	Predominant evidence source category/safety
Nitrous Oxide (N_2_O)	Inhalation: typically 30%–50% N_2_O with oxygen, titrated to clinical effect	Mild anxiety, cooperative patients, minimal gag reflex	Rapid onset and recovery, titratable, minimal systemic effects	Ineffective for severe anxiety; contraindicated in respiratory compromise or nasal obstruction (11, 28, 29)	A/B/C sources; favorable safety profile, standard monitoring
Oral Midazolam	Pediatric oral: 0.3–0.5 mg/kg in cited dental studies; dose individualized according to patient and protocol	Mild to moderate anxiety, pediatric patients	Easy administration, effective anxiolysis	Variable absorption, delayed onset, paradoxical reactions in some children (3, 6, 12, 13)	B; monitoring/rescue capability
Intranasal Midazolam	Pediatric intranasal: 0.3–0.5 mg/kg in cited studies; patient- and protocol-specific	Moderate anxiety, uncooperative children, short procedures	Rapid onset, avoids IV access	Nasal irritation, bitter taste, variable acceptance (4, 7, 21)	A; monitoring/rescue capability
IV Midazolam	IV: slow incremental administration, individually titrated to clinical effect according to age, clinical status, and concomitant medications	Titratable procedural sedation in appropriately selected patients; achieved depth depends on dose, titration, co-administered agents, and patient response.	Precise titration, rapid onset, predictable effect	Requires IV access and monitoring, risk of oversedation (14, 36, 37)	C; airway-rescue capability
Dexmedetomidine	IN/IV: weight- and protocol-specific dosing; regimens vary by population, route, and intended sedation depth	Sedation in selected patients, including behaviorally complex cases; achieved depth is dose-, route-, and protocol-dependent.	Minimal respiratory depression, stable hemodynamics	Slow onset, prolonged recovery, risk of bradycardia (8, 30, 32, 33)	A; cardiorespiratory monitoring
Dexmedetomidine + Ketamine	Intranasal, Buccal, or IV: variable dosing	Severe anxiety, uncooperative children, ASD patients	Synergistic sedation, effective for challenging behaviors	Requires monitoring; risk of hypersalivation and emergence reactions (30, 31, 33)	A; standard monitoring
Propofol ( ± adjuncts)	IV: individually titrated according to clinical response, intended sedation depth, patient characteristics, and institutional protocol	Deep sedation, surgical procedures	Rapid onset and recovery, titratable	Requires anesthetist, airway support, risk of hypotension (22, 36)	A/C; advanced monitoring/airway rescue
Remimazolam	IV: titrated dosing	Procedural sedation in adults, complex dental procedures	Predictable effect, rapid recovery, less respiratory depression	High cost, limited pediatric data (22, 23)	A/B; limited dental evidence
Flumazenil (Reversal Agent)	Adult procedural-sedation reversal: initial 0.2 mg IV, with additional 0.2 mg doses as needed up to a total of 1 mg; pediatric dosing is age-/weight- and protocol-specific	Reversal of benzodiazepine oversedation (e.g., midazolam)	Rapid reversal, improves safety	Risk of re-sedation due to shorter half-life; use cautiously (34, 35)	A; monitor for re-sedation

Evidence-source category indicates the predominant category among references supporting each intervention and is used for descriptive organization only; it does not represent formal evidence-quality or certainty grading. Where no category predominated, the relevant categories are shown.

Table 4 provides a structured, scenario-based framework to guide dental practitioners and sedationists in selecting appropriate sedation strategies based on patient-specific clinical presentations. For appropriately selected patients with mild anxiety and cooperative behavior, nitrous oxide–oxygen inhalation is a well-supported minimal-sedation option because of its titratability, rapid onset and recovery, and favorable safety profile (11, 13, 28, 29). In cases of moderate anxiety, moderate gag reflex, or partial cooperation, oral or intranasal midazolam, often in combination with nitrous oxide, provides reliable anxiolysis (3, 4, 6, 7, 13). For older children, adolescents, and adults presenting with high anxiety or dental phobia, intravenous midazolam allows superior titratability and amnesia when accompanied by full physiological monitoring (14, 35, 37). Propofol-based deep sedation may provide rapid onset, titratability, and predictable recovery in appropriately selected patients; however, its use requires appropriately trained and credentialed personnel, continuous monitoring appropriate to deep sedation, immediate airway-rescue and positive-pressure ventilation capability, and compliance with institutional and jurisdiction-specific requirement (22, 32, 36). For complex cases involving severe anxiety, autism spectrum disorder, or significant gag reflexes, a dexmedetomidine–ketamine combination may be used under anesthetic supervision, though with heightened risks such as bradycardia, hypersalivation, and emergence reactions (30, 31, 33). Table 5 summarizes emergency events relevant to dental sedation and procedural care, together with their immediate management and key preparedness considerations. Table 6 presents an evidence-informed conceptual framework for individualized sedation selection in dental practice. The framework emphasizes selecting the least invasive sedation technique likely to achieve the desired clinical objective while recognizing that patients may enter at different levels according to their age, medical and ASA physical status, airway risk, anxiety and cooperation, procedural requirements, previous sedation response, clinician competence, and available monitoring and rescue resources. Rather than serving as a mandatory sequential escalation pathway or prescribing algorithm, the framework is intended as a conceptual clinical aid to support individualized, evidence-informed decision-making consistent with applicable professional and regulatory requirements.

**Table 4:** Scenario-based dental sedation strategies: indicative dosing, monitoring, emergency preparedness, and evidence sources.

Clinical scenario	Sedation strategy	Indicative dose/route	Monitoring	Emergency preparedness	Evidence sources/safety context
Mild anxiety; cooperative patient	Nitrous oxide–oxygen	Inhaled: N_2_O 30%–50%, titrated to clinical effect	Clinical observation; SpO_2_ and pulse according to sedation depth and patient risk	100% O_2_, suction, basic airway equipment	A/B/C sources; standard monitoring (11, 13, 28, 29)
Moderate anxiety, gag reflex, or reduced cooperation	Midazolam ± N_2_O, where appropriate	Pediatric oral: 0.3–0.5 mg/kg; pediatric IN: 0.3–0.5 mg/kg in cited studies; individualized to patient and protocol	SpO_2_, HR, and ventilation appropriate to sedation depth; capnography where indicated or required	O_2_, suction, age-appropriate airway equipment; flumazenil available	A-predominant sources; monitoring/rescue capability (3, 4, 6, 7, 13)
High anxiety/dental phobia requiring titratable moderate sedation	IV midazolam ± N_2_O, where appropriate	IV: slow incremental administration, individually titrated to clinical effect	SpO_2_, HR, BP, and ventilation; capnography where indicated or required	O_2_, suction, airway equipment; flumazenil available; immediate rescue capability	C-predominant sources; airway-rescue capability (14, 36, 37)
Selected patients requiring deep sedation for complex or prolonged procedures	Propofol-based deep sedation	IV: individually titrated according to clinical response, intended sedation depth, patient characteristics, and institutional protocol	Continuous SpO_2_, ECG, BP, capnography, and clinical observation	Immediate airway rescue and positive-pressure ventilation; advanced resuscitation equipment and emergency medications	A-predominant sources; advanced monitoring/airway rescue (20, 22, 27, 32, 36)
Selected paediatric or behaviourally complex patients requiring specialized sedation	Dexmedetomidine- and/or ketamine-based regimens	IN/IV: weight- and protocol-specific dosing according to selected regimen	Continuous cardiorespiratory and sedation-depth monitoring; airway readiness	O_2_, suction, age-appropriate airway/resuscitation equipment; immediate rescue capability	A sources; specialized monitoring/rescue (30, 31, 33)

Doses are indicative and should be individualized according to patient characteristics, intended sedation depth, clinical response, and applicable protocols. Pediatric and adult doses should not be extrapolated interchangeably. Monitoring and rescue capability should match the intended and reasonably foreseeable sedation depth (16–20, 27). A–C denote evidence-source categories for narrative organization and do not represent formal certainty-of-evidence grades.

ASA, American Society of Anesthesiologists; BP, blood pressure; ECG, electrocardiography; HR, heart rate; IN, intranasal; IV, intravenous; N_2_O, nitrous oxide; O_2_, oxygen; SpO_2_, peripheral oxygen saturation.

**Table 5:** Emergency events relevant to dental sedation and procedural care: recognition, immediate management, and preparedness.

Emergency	Immediate management	Medication/intervention	Dose/route	Key consideration
SEDATION-RELATED ADVERSE EVENTS				
Airway obstruction/apnea	Reposition airway, suction, O_2_, assisted ventilation	100% O_2_; bag-mask ventilation	—	Escalate airway support if ventilation is not restored
Bradycardia	Assess oxygenation, ventilation, and perfusion	Atropine, if indicated	Age-/weight-specific	Treat reversible causes; adult/pediatric algorithms differ
Hypotension	Supine position, IV access, assess cause	IV crystalloid ± vasoactive therapy	Clinical/protocol-specific	Identify sedation-related or other underlying causes
MEDICATION/REVERSAL-RELATED EMERGENCIES				
Benzodiazepine oversedation	Airway support, O_2_, assisted ventilation	Flumazenil	Age-/protocol-specific IV titration	Monitor for re-sedation
Opioid respiratory depression	Airway support, O_2_, assisted ventilation	Naloxone	Age-/protocol-specific IV/IM/IN titration	Repeat dosing may be required
Local anesthetic systemic toxicity (LAST)	Stop local anesthetic; airway support; treat seizures	20% IV lipid emulsion	Established LAST protocol	Immediate advanced resuscitation/transfer
GENERAL MEDICAL EMERGENCIES DURING DENTAL CARE				
Anaphylaxis	Stop trigger, activate EMS, airway support, high-flow O_2_	IM epinephrine	Age-/weight-specific	First-line treatment; repeat according to guideline
Seizure	Protect patient, airway support, O_2_	Midazolam, if indicated	Route- and age-/weight-specific	EMS for prolonged/recurrent seizure
Hypoglycemia	Check glucose; treat promptly	Oral glucose/IV glucose/IM glucagon	Age-/weight-specific	Route depends on consciousness and IV access
Cardiac arrest	CPR, EMS, AED/defibrillation	Resuscitation therapy	Adult/pediatric life-support protocol	Do not delay CPR or defibrillation
ANESTHESIA-SPECIFIC EMERGENCY				
Malignant hyperthermia	Stop triggering agents, 100% O_2_, cooling, EMS	Dantrolene	According to current MH protocol	Relevant primarily when recognized MH-triggering anesthetic agents are used; not a routine complication of the principal conscious-sedation agents reviewed

Emergency management should follow current adult or pediatric resuscitation and condition-specific guidance, with age- and weight-appropriate drugs and equipment. Oxygen, suction, bag-mask ventilation, airway equipment, AED, relevant reversal agents, and emergency medications should be immediately available (16–20, 27–35).

AED, automated external defibrillator; CPR, cardiopulmonary resuscitation; EMS, emergency medical services; IM, intramuscular; IN, intranasal; IV, intravenous; LAST, local anesthetic systemic toxicity; MH, malignant hyperthermia; O_2_, oxygen.

**Table 6:** Evidence-informed conceptual sedation framework for dental practice.

Sedation category	Typical clinical context	Potential approach	Monitoring and safety requirements	Evidence category
Level 1: Minimal sedation	Mild anxiety; cooperative patient	Nitrous oxide–oxygen, titrated to clinical effect	Clinical observation and physiological monitoring appropriate to patient risk and sedation depth; recovery to baseline before discharge	A
Level 2: Non-IV pharmacological sedation	Moderate anxiety, gag reflex, or reduced cooperation	Oral or intranasal midazolam ± nitrous oxide–oxygen, where appropriate	SpO_2_, HR, and ventilation monitoring appropriate to sedation depth and patient risk; capnography where indicated or required; airway and rescue equipment available	A–B
Level 3: Titrated moderate sedation	Selected patients requiring titratable moderate sedation	IV midazolam ± nitrous oxide–oxygen, where appropriate	SpO_2_, HR, BP, and ventilation monitoring; capnography where indicated or required; immediate airway-rescue capability	A–B
Level 4: Advanced/deep sedation	Selected patients requiring deep sedation for complex or prolonged procedures	Propofol-based sedation or other advanced techniques in an appropriately equipped setting	Continuous SpO_2_, ECG, BP, and capnography; appropriately trained/credentialed personnel; immediate airway rescue and positive-pressure ventilation capability	A–B; setting-dependent
Specialized/alternative approaches	Selected pediatric, behaviorally complex, or other appropriate patients	Dexmedetomidine- and/or ketamine-based regimens	Appropriate cardiorespiratory and sedation-depth monitoring; trained personnel and immediate rescue capability; protocol- and setting-dependent	A–B; specialized/setting-dependent

This framework is an evidence-informed conceptual clinical aid and should not be interpreted as a mandatory sequential escalation pathway or validated prescribing algorithm. Patients may enter at different levels or require specialized/alternative approaches according to age, medical and ASA physical status, airway risk, anxiety and cooperation, procedural requirements, previous sedation response, clinician competence, facility resources, and applicable professional and regulatory requirements. MDAS, Frankl, and GSI scores may support assessment but should not independently determine drug selection, route, dose, or sedation depth (16–20, 27). Monitoring, personnel, credentialing, permitted sedation depth, and drug-use requirements should follow applicable national or local professional and regulatory standards.

ASA, American Society of Anesthesiologists; BP, blood pressure; ECG, electrocardiography; GSI, gagging severity index; HR, heart rate; IN, intranasal; IV, intravenous; MDAS, modified dental anxiety scale; SpO_2_, peripheral oxygen saturation.

Across the included evidence, nitrous oxide-oxygen and midazolam-based techniques had the broadest support for commonly used minimal-to-moderate dental sedation, although route, dose, and patient suitability varied by age and clinical context. Evidence also supported the use of advanced sedative agents and multidrug regimens in selected populations; however, their applicability was more dependent on provider expertise, intended sedation depth, monitoring capability, and clinical setting. Propofol-based deep sedation and specialized multidrug regimens require advanced training, airway-rescue capability, appropriate physiological monitoring, and compliance with applicable professional and regulatory standards. The resulting sedation framework therefore emphasizes individualized selection rather than automatic escalation according to anxiety or behavioral scores. The framework begins with consideration of the least invasive technique likely to achieve the clinical objective while recognizing that patients may enter at different levels or require alternative approaches based on medical status, airway risk, procedural complexity, previous sedation response, and available clinical resources. The framework should be interpreted as an evidence-informed conceptual aid rather than a validated prescriptive algorithm.

## Discussion

This review highlights the importance of an individualized, evidence-informed approach to dental sedation that integrates patient assessment, pharmacological characteristics, procedural requirements, monitoring, and emergency preparedness. Sedation exists along a continuum from minimal to deep sedation, and selection of an appropriate technique should be based on the patient's age, medical and ASA physical status, airway risk, anxiety and cooperation, previous sedation response, procedural complexity and duration, clinician competence, and available monitoring and rescue resources (16, 19, 27). Importantly, sedation depth is not determined by the drug alone but by dose, route, titration, drug combinations, patient response, and procedural context. Because unintended progression to deeper sedation may occur, monitoring and rescue capability should correspond to the intended and reasonably foreseeable sedation depth, with practitioners able to rescue patients from a deeper-than-intended level (16–20, 27). Assessment tools such as the Modified Dental Anxiety Scale (MDAS), Frankl Behavior Rating Scale, and Gagging Severity Index (GSI) may assist in characterizing anxiety, cooperation, and gag-reflex severity; however, they should support rather than independently determine drug selection, route of administration, or sedation depth.

Nitrous oxide–oxygen remains a well-supported option for minimal sedation in appropriately selected patients because of its rapid onset and recovery, titratability, and favorable safety profile (11, 28, 29). It is particularly useful in cooperative patients with mild-to-moderate anxiety and in selected pediatric populations. When additional pharmacological support is required, midazolam may be administered through oral, intranasal, or intravenous routes according to patient characteristics, clinical requirements, and provider competence (3, 4, 7, 13, 14). Oral and intranasal administration may be useful in selected pediatric or behaviorally challenging patients, whereas intravenous administration provides greater titratability in appropriately selected older patients and adults (3, 4, 7, 13, 14, 36, 37). These approaches should not be regarded as mandatory sequential steps; patients may require different techniques according to individual clinical circumstances. Remimazolam is a newer benzodiazepine with potential advantages for procedural sedation, including rapid onset and recovery and titratability. However, its evidence base in dental sedation remains comparatively limited, particularly in pediatric populations, and current findings should therefore be regarded as emerging rather than equivalent to the more established evidence supporting midazolam or nitrous oxide. Further dental-specific studies are needed before its role in routine dental sedation can be clearly established (22, 23).

Advanced or deep sedation requires substantially greater clinical expertise, monitoring, and rescue capability. Propofol-based techniques provide rapid onset, titratability, and predictable recovery but may produce respiratory depression, airway obstruction, and cardiovascular instability (20, 22, 27, 32, 36). Accordingly, deep sedation should be undertaken only by appropriately trained and credentialed personnel in suitably equipped settings, with continuous physiological monitoring appropriate to the intended and reasonably foreseeable sedation depth and immediate capability for airway rescue and positive-pressure ventilation (16–20, 27). Selection for deep sedation should therefore be based on comprehensive patient, airway, procedural, and setting-specific assessment rather than anxiety or behavioral scores alone.

Dexmedetomidine- and ketamine-based regimens represent specialized or alternative sedation approaches rather than a final step in a universal escalation pathway. Dexmedetomidine offers sedation with relatively limited respiratory depression but may be associated with bradycardia, hypotension, and prolonged recovery, whereas ketamine provides dissociative sedation and analgesia but may cause hypersalivation and emergence reactions (30–33). Combination regimens may be useful in selected pediatric or behaviorally complex patients, including some patients with autism spectrum disorder or substantial cooperation difficulties; however, heterogeneity in dosing, routes, populations, and clinical settings limits the development of a single standardized protocol (30–33). Their use should therefore depend on appropriate patient selection, clinician expertise, monitoring capability, and institutional protocols.

Drug interactions and patient-specific pharmacological factors are also important considerations in sedation planning. Midazolam metabolism may be affected by cytochrome P450 3A4 inhibitors, including macrolide antibiotics and azole antifungals, potentially increasing or prolonging sedation (36, 38). The sedative and respiratory effects of propofol may be potentiated when combined with opioids or other central nervous system depressants (22, 36). Nitrous oxide also requires appropriate patient selection, particularly where vitamin B12 deficiency or prolonged/repeated exposure may increase the risk of neurological complications (23). Medication review, comorbidity assessment, and dose individualization are therefore essential components of pre-sedation evaluation.

Monitoring requirements should correspond to the intended and reasonably foreseeable depth of sedation, patient risk, and applicable professional standards. Pulse oximetry provides important information regarding oxygenation but does not directly measure ventilation; therefore, ventilation assessment, including capnography where indicated or required, is particularly important as sedation depth increases or when patient and procedural factors increase the risk of respiratory compromise (16–20, 27). Moderate and deep sedation additionally requires appropriate cardiovascular monitoring and personnel capable of recognizing and managing unintended progression to a deeper level of sedation. Safe practice also requires immediate access to oxygen, suction, age-appropriate airway equipment, reversal agents where applicable, and resuscitation resources appropriate to the deepest level of sedation that may reasonably occur (16–20, 34–36). Although core safety principles such as appropriate patient assessment, monitoring, and rescue capability are broadly applicable, credentialing standards, permitted sedation depth, personnel and monitoring requirements, and drug availability vary across jurisdictions. Clinical application of this framework should therefore follow applicable national or local professional and regulatory requirements.

Emergency preparedness depends not only on the availability of drugs and equipment but also on team competency, accessibility, and maintenance of emergency resources. Recent real-world evidence has identified gaps in emergency training and essential equipment availability in dental clinics, reinforcing the importance of regular team training appropriate to the sedation level, defined emergency roles, mock drills, documented team competency, and routine checks of emergency equipment and medication expiry dates (16–20, 39). The evidence synthesized in this review supports a flexible, individualized sedation framework rather than a rigid, score-based or mandatory sequential escalation pathway. Nitrous oxide–oxygen and midazolam-based techniques have broad support for appropriately selected patients requiring minimal-to-moderate sedation, whereas advanced agents and multidrug regimens require greater expertise, monitoring, and setting-specific resources (3, 4, 7, 11, 13, 14, 20, 22, 28–33, 36, 37). The least invasive technique likely to achieve the clinical objective should generally be considered while recognizing that sedation selection is not necessarily sequential and that some patients may appropriately require alternative or advanced approaches from the outset following comprehensive assessment.

Several limitations should be considered when interpreting the proposed framework. This structured, evidence-informed narrative review was not a prospectively registered systematic review, and heterogeneity across populations, sedation protocols, outcomes, and clinical settings precluded quantitative synthesis. Formal study-level risk-of-bias or methodological-quality appraisal was not performed; therefore, the A–C classification should be regarded only as an organizational approach and not as formal evidence-quality or certainty grading. Some recommendations incorporated transferable evidence from non-dental procedural sedation. Finally, the proposed framework has not been prospectively validated and should be regarded as a conceptual clinical aid that complements professional guidelines, clinical judgment, credentialing requirements, and jurisdiction-specific regulations (16–20, 27).

## Conclusion

This evidence-informed narrative review indicates that no single sedative drug or administration route is universally optimal for all dental patients. Instead, sedation should be individualized according to patient age, medical and ASA status, airway risk, anxiety and cooperation, procedural requirements, intended sedation depth, and available clinical expertise and resources. Nitrous oxide–oxygen and midazolam-based techniques remain well-supported options for appropriately selected patients requiring minimal-to-moderate sedation, whereas advanced or deep sedation requires appropriately trained and credentialed personnel, appropriate monitoring, and immediate airway-rescue capability. Dexmedetomidine- and ketamine-based regimens may provide specialized alternatives in selected patients and clinical settings.

The proposed framework should be regarded as an evidence-informed conceptual clinical aid rather than a rigid sequential or universally prescriptive algorithm. Its application should remain consistent with professional guidance, clinician competence, institutional protocols, facility resources, and applicable regulatory requirements. Further prospective research is needed to validate standardized patient-selection pathways, dosing strategies, and clinical outcomes across diverse dental populations and settings.
