# Supplementary material for: An updated evidence-informed framework for dental sedation: drugs, routes, patient selection, monitoring, and emergency preparedness
Source: Front Oral Health. 2026 Sep 16;7:1915718. doi: 10.3389/froh.2026.1915718 (PMC13623722; doi:10.3389/froh.2026.1915718)
Supplement: Supplementary file 1 [file Table1.docx]

Table S1. Complete Database-Specific Search Strategies

| Database | Exact search strategy | Limits | Final search |
| --- | --- | --- | --- |
| PubMed/MEDLINE | Exact query | English; date range | September 2025 |
| Scopus | Exact query | English; date range | September 2025 |
| Web of Science | Exact query | English; date range | September 2025 |
| Cochrane Library | Exact query | Date range | September 2025 |
